# Hemopexin as an Inhibitor of Hemolysis-Induced Complement Activation

**DOI:** 10.3389/fimmu.2020.01684

**Published:** 2020-07-31

**Authors:** Victoria Poillerat, Thomas Gentinetta, Juliette Leon, Andreas Wassmer, Monika Edler, Carine Torset, Dandan Luo, Gerald Tuffin, Lubka T. Roumenina

**Affiliations:** ^1^Centre de Recherche des Cordeliers, INSERM, Sorbonne Université, Université de Paris, Paris, France; ^2^CSL Behring AG, Bern, Switzerland; ^3^CSL Behring, King of Prussia, PA, United States

**Keywords:** hemopexin, heme, hemolysis, complement, kidney injury, C3

## Abstract

Hemopexin is the main plasmatic scavenger of cell-free heme, released in the context of intravascular hemolysis or major cell injury. Heme is indispensable for the oxygen transport by hemoglobin but when released outside of the erythrocytes it becomes a danger-associated molecular pattern, contributing to tissue injury. One of the mechanisms of pro-inflammatory action of heme is to activate the innate immune complement cascade. Therefore, we hypothesized that injection of hemopexin will prevent hemolysis-induced complement activation. Human plasma-derived hemopexin is compatible with the heme clearance machinery of the mice. 100 or 500 mg/kg of hemopexin was injected in C57Bl/6 mice before treatment with phenylhydrazine (inducer of erythrocytes lysis) or with PBS as a control. Blood was taken at different timepoints to determine the pharmacokinetic of injected hemopexin in presence and absence of hemolysis. Complement activation was determined in plasma, by the C3 cleavage (western blot) and in the kidneys (immunofluorescence). Kidney injury was evaluated by urea and creatinine in plasma and renal NGAL and HO-1 gene expression were measured. The pharmacokinetic properties of hemopexin (mass spectrometry) in the hemolytic mice were affected by the target-mediated drug disposition phenomenon due to the high affinity of binding of hemopexin to heme. Hemolysis induced complement overactivation and signs of mild renal dysfunction at 6 h, which were prevented by hemopexin, except for the NGAL upregulation. The heme-degrading capacity of the kidney, measured by the HO-1 expression, was not affected by the treatment. These results encourage further studies of hemopexin as a therapeutic agent in models of diseases with heme overload.

## Introduction

In physiological conditions heme is compartmentalized inside the cells and serves as an indispensable cofactor for aerobic life, by its interaction with conventional heme-binding proteins, such as hemoglobin, myoglobin and cytochromes. Nevertheless, it becomes a danger associated molecular pattern, when released in the circulation or in tissues during intravascular hemolysis [red blood cells (RBC) lysis during sickle cell disease (SCD), hemolytic uremic syndrome (HUS), malaria, transfusion reactions, etc.] or rhabdomyolysis (crush syndrome, muscle damage as in car accidents, natural cataclysms, or military trauma) (1). Heme triggers inflammation by activating immune and endothelial cells (EC) or plasma systems, such as the complement cascade, coagulation, or inducing antibody polyreactivity (2–4). In physiological conditions excess of cell-free heme is scavenged by its natural binding protein hemopexin (Hpx).

Hpx is a liver-produced plasma glycoprotein (0.5–1.15 g/l) of 60 kDa. Hpx binds free heme with a very high affinity (Kd <10^−13^ M) (5, 6), which makes it virtually irreversible. The heme:Hpx complex binds to CD91/LRP1 and is endocytosed (7). Part of Hpx may be recycled, but the majority is degraded, creating acquired Hpx deficiency in case of massive hemolysis (6). Hpx-knockout mice presented severe renal injury upon phenylhydrazine (PHZ triggered hemolysis), contrary to the WT mice (8). Moreover, Hpx deficiency promoted acute kidney injury in sickle mice under hemolytic stress, which was blocked by pre-treatment with purified Hpx (9). Injection of heme in SCD mice induce stasis (10, 11), cardiovascular injury and cardiomyocytes alteration (12–14), all of which have been prevented by pre-treatment with Hpx. Heme-carrying erythrocyte microparticles from SCD patients, injected in a SCD mice, induced kidney vaso-occlusion and endothelium injury, which was also prevented by Hpx administration (15, 16). Hpx also prevents the activation of the pro-inflammatory complement system in the kidneys of hemolytic mice (PHZ model) and *in vitro*, in serum and on endothelial cells. Moreover, it prevented the complement deposition on endothelial cells, incubated with serum from SCD patients (17). This complement activation plays a key role in the organ injury in SCD and in hemolytic mice, since C3 deficiency or complement blockade alleviate the vaso-occlusion and the kidney and liver damage, respectively (18, 19).

Taken together, these examples demonstrate that replenishing the Hpx pool is a potential promising therapeutic strategy to avoid the heme-mediated toxicity. In order to be tested as a therapeutic agent in pre-clinical models, the pharmacokinetic, and the active concentrations of Hpx have to be evaluated in the context of an intravascular hemolysis. Here we demonstrate that the pharmacokinetic properties of Hpx in the hemolytic mice were affected by the target-mediated drug disposition phenomenon. The dose of 100 mg/kg is well tolerated and sufficient to prevent the hemolysis-induced complement activation.

## Method

### Animal Experimentation

Experimental protocols were approved by Charles Darwin ethical committee (Paris, France) and of French Ministry of Agriculture (Paris, France) number #3764 201601121739330 v3. All experiments were conducted in accordance with the recommendations for the care and use laboratory animal.

**Figure 1:**
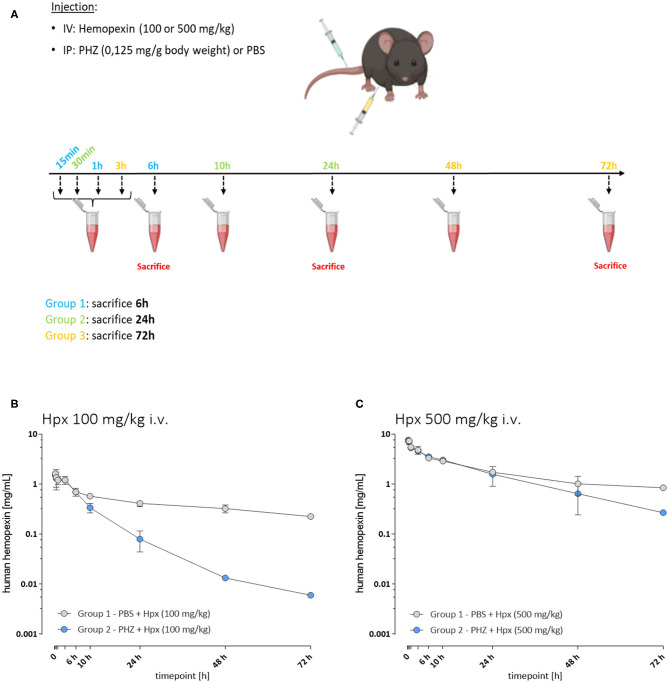
The pharmacokinetic of hemopexin in hemolytic mice is affected by target-mediated drug disposition phenomenon. **(A)** Protocol to study pharmacokinetic of Hpx. Three groups of mice were injected with PHZ and Hpx and bled as follows: Group 1 (15 min, 1 and 6 h), Group 2 (30 min, 10 and 24 h), and Group 3 (3, 48, and 72 h). **(B,C)** *In vivo* Hpx exposure in presence and absence of induced hemolysis. **(B)** Mean ± SD plasma concentration vs. time plotted for human hemopexin administered to mice (100 mg/kg i.v.; *n* = 3/timepoint). In presence of PHZ (0.125 mg/g weight, blue circles) or control (PBS, gray circles). Pharmacokinetic parameter estimates are shown in Table 1. **(C)** Mean ± SD plasma concentration vs. time plotted for human hemopexin administered to mice (500 mg/kg; *n* = 3/timepoint). In presence of PHZ (0.125 mg/g weight, blue circles) or control (PBS, gray circles). Pharmacokinetic parameter estimates are shown in Table 1.

A first experiment was performed to determine the pharmacokinetic properties of Hpx in hemolytic mice (Figure 1A). Three groups of mice were injected in i.v. with 100 mg/kg of human plasma derived Hpx (CSL Behring) and three other groups with 500 mg/kg of Hpx. An i.p. PHZ injection (0.125 mg/g body weight) was performed immediately after Hpx administration. The mice from each Group 1 were bled at 15 min, 1 and 6 h. They were sacrificed at 6 h. Each Group 2 was bled at 30 min, 10 and 24 h and sacrificed at 24 h. Each Group 3 was bled at 3, 48, and 72 h followed by a sacrifice at 72 h. The bleeding schema is given in Supplementary Table 1.

**Figure 2:**
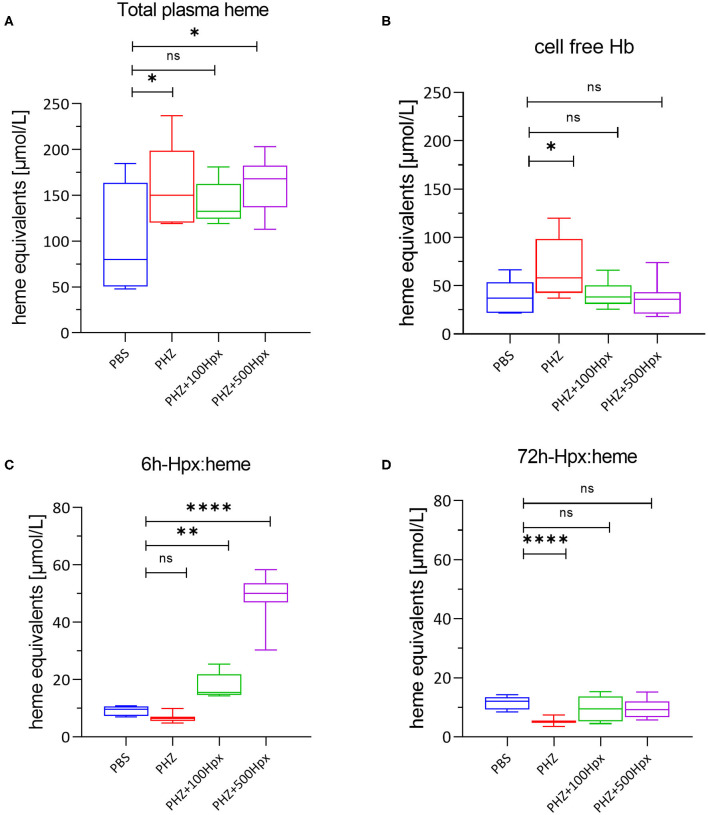
Heme scavenging upon PHZ induced hemolysis by hemopexin. **(A)** Mean total heme plasma concentration at 6 h, **(B)** mean cell free hemoglobin at 6 h and **(C)** hemopexin:heme complexes at 6 h, and **(D)** 72 h shown for each group [(1) PBS; *n* = 7, (2) PHZ, (3) PHZ + 100 mg/kg, and (4) PHZ + 500 mg/kg; *n* = 10]. Box and whiskers plots represent means ± Min to Max. ****p < 0.0001, **p < 0.01, and *p < 0.05 comparisons to PBS treated, Two-way ANOVA Kruskal Wallis test, ns, not significant.

A second experiment aimed to evaluate the complement inhibition capacity of Hpx and its impact on complement activation and renal function. Three groups of 8-week-old C56BL/6 male mice (*n* = 10) were pretreated with human Hpx (CSL Behring) in i.v. with 100 or 500 mg/kg or equivalent volume of PBS (0 mg/kg) immediately before i.p. PHZ injection (0.125 mg/g body weight) (Figure 2A). A control group of 8 mice received two injections of PBS, corresponding to the volume of Hpx and PHZ. All mice were sacrificed by cervical dislocation, 6 or 72 h after Hpx administration. Whole blood was collected 3 days before experimentation and at day 1 into microtubes filled with 2 μL of heparin (Heparine Choay® 5000 ui/ L Sanofi) by venipuncture in the cheek. Microtubes were centrifuged at 604 g for 10 min at room temperature to separate plasma. Kidneys were harvested for immunofluorescence (IF) and gene expression analyses. Plasma and organs were directly frozen in liquid nitrogen and stored at −80°C.

### Quantification of Human Hpx in Animal Plasma by LC/MS

Ten microliters of plasma sample were placed into a clean Eppendorf tube followed by the addition of 80 μL MeOH to precipitate the protein. The methanol was removed after centrifugation and the pellet was air-dried and afterwards re-suspended in 50 mM NH_4_HCO_3_/0.16% ProteaseMAX containing a heavy-isotope labeled peptide, which is specific for human Hpx and is used as internal standard. After incubation at 56°C/550 rpm for 45 min the samples were reduced by adding 0.5 M DTT (56°C/550 rpm for 20 min). The samples were then alkylated by addition of 0.5 M IAA and incubation for 20 min at RT protected from light. Tryptic digestion was carried out at 37°C/550 rpm and stopped after 3 h by addition of formic acid. After centrifugation the samples were separated immediately on a C18 column (AdvanceBio Peptide Mapping, 2.1 × 150 mm). The measurements were conducted using an Agilent 1290 Infinity II – 6550 iFunnel QTOF LC-MS system.

Data was analyzed by calculating the peak area of the analyte and the internal standard using Agilent MassHunter Quant software. A standard curve was created in Agilent MassHunter Quant by plotting the average response ratio of analyte to internal standard against concentration for each standard sample. The analyte concentration in the plasma samples was backcalculated using the standard curve equation.

### Preparation of Heme

Hemin (Frontier Scientific) was dissolved in 10 mL NaOH (100 mmol/L) at 37°C. The pH of the solution was adjusted to pH 7.8 using phosphoric acid. The solution was sterile-filtered (0.22 μm) and used immediately.

### Total Heme in Mouse Plasma

Total plasma heme concentration in mouse plasma was determined according manufacturer's protocols using the QuantiChrom^TM^ Heme Assay Kit (BioAssay Systems). Briefly, 50 μL of sample (diluted in water 1:2) was placed into a 96-well plate. Assay reagent (200 μl per well) was added and incubated for 5 min at room temperature. Absorbance at λ_400_ nm was measured using a microplate reader (Synergy BioTek). Heme concentration was determined by comparison to a hemin standard curve (hemin preparation see above).

### Detection of Heme:Hpx Complexes in Mouse Plasma

Fifty microliters of plasma sample were placed into a clean Eppendorf tube followed by the addition of 150 μL Buffer A (Multiple Affinity Removal Systems, Agilent). In a first chromatography step high abundant mouse proteins were depleted and carried out according to the manufacturer's protocol on an Ultimate 3000SD HPLC attached to two LPG-3400SD quaternary pumps and a photodiode array detector (DAD) (ThermoFisher). Briefly, the diluted plasma sample was injected onto a multi affinity removal column depleting mouse albumin, IgG, and Transferrin (Mouse-3, 4.6 × 50 mm, Agilent) and separated with Buffer A (Multiple Affinity Removal Systems, Agilent) as the mobile phase at a flow rate of 0.25 mL/min. Depleted plasma was collected into a fresh HPLC vial and re-injected and separated on a Diol-300 (3 μm, 300 × 8.0 mm) column (YMC Co., Ltd.) with PBS, pH 7.4 (Bichsel) as the mobile phase at a flow rate of 1 mL/min. For all samples two wavelengths were recorded (λ = 280 nm and λ = 414 nm). The amount of Heme:Hpx complexes was determined by calculating the peak area of the complex (9 min retention time). Values from depleted plasma samples were interpolated by generating a standard curve based on peak area and plotted against the concentrations.

### Plasma Hb Measurements

Hp-bound and unbound fractions of Hb (cell free Hb) were determined by SEC–high-performance liquid chromatography (SEC-HPLC) using an Ultimate 3000SD HPLC attached to a LPG-3400SD quaternary pump and a photodiode array detector (DAD) (ThermoFisher). Plasma samples and Hb standards were separated on a Diol-120 (3 μm, 300 × 8.0 mm) column (YMC Co., Ltd.) with PBS, pH 7.4 (Bichsel) as the mobile phase at a flow rate of 1 mL/min. For all samples two wavelengths were recorded (λ = 280 nm and λ = 414 nm). Bound and unbound Hb in plasma was determined by calculating the peak area of both peaks (6 min retention time for Hb:Hp, 8 min retention time for cell free Hb). Values from plasma samples were interpolated by generating a standard curve based on peak area and plotted against the concentrations.

### Pharmacokinetic (PK) Analysis

Four groups were evaluated: (I) PBS + Hpx (100 mg/kg i.v.), (II) PHZ + Hpx (100 mg/kg i.v.), (III) PBS + Hpx (500 mg/kg i.v.), and PHZ + Hpx (500 mg/kg i.v.) (group size *n* = 3/timepoint).

Hpx PK in PBS vs. PHZ treated mice were conducted via non-compartmental analysis (NCA) using Phoenix WinNonlin version 7.4 (Certara, St. Louis, MO, USA). Linear up-log down method was used for area under the concentration curve (AUC) calculation. Besides directly observed maximum concentration (C_max_) and AUC_0−72h_, other derived PK parameters including area under the concentration curve till infinity (AUC_inf_), clearance (CL), volume at steady state (Vss), and half-life (T1/2) were reported.

### Evaluation of the Kidney and Liver Function

Kidney function was evaluated by blood urea nitrogen (urea) and creatinine, measured by a colorimetric analysis using Konelab Clinical Chemistry Analyzers in the Renal Function Exploration platform of the Cordeliers Research Center. ALT was measured using Olympus AU400 multiparameter equipment on the biochemistry platform in Hospital Bichat (Centre Recherche sur l'Inflammation-Paris).

### Immunofluorescence

Six micrometer thick frozen kidney sections were cut with Cryostat at −20°C (Leica AS-LMD, Leica Biosystem) and fixed in acetone on ice for 10 min. The primary antibody was C3b/iC3b (rat anti-mouse, Hycult biotech, HM1065, 1 μg/ml) and CD31 (rat anti-mouse, Abcam Ab7388, 2 μg/mL). Staining was revealed by Donkey anti-rabbit AF647 (Thermoscientific, A21447, 5 μg/mL) and chicken anti-rat AF488 (Thermoscientific, A21470, 5 μg/mL). Slides were scanned by Axio Scan™ Z1 (Zeiss, Oberkochen, Germany). Images were analyzed using Zen lite software (Zeiss). C3 and CD31 staining were quantified using HALO® (Indica Labs) software.

### Gene Expression Analysis

Frozen kidneys were sectioned at 30 μm with Cryostat at −20°C (Leica AS-LMD, Leica Biosystem). Twenty sections were recovered and homogenized in the tubes with 200 μL of 1-Thioglycerol/Homogenization Solution (Maxwell® 16 LEV simplyRNA Tissue Kit Promega AS1280). The quality and quantity of mRNA were evaluated with the Agilent 2100 bioanalyzer using the Agilent TNA 6000 NanoKit, followed by retro-transcription to cDNA. All RNA Integrity Numbers superior to 7 were retained for reverse transcription in cDNA. Gene markers of early kidney injury [LCN2 (Lcn2-Mm01324470_m1)], and for cytoprotection [HO-1 (Hmox1-Mm00516005_m1)] was analyzed with SDS 2.1® software (ThermoFisher), after normalization on actin (Actb_Mm02613580_g1) housekeeping gene expression. The gene expression for the PHZ-treated mice was expressed as fold change compared to the gene expression from the pool of the PBS treated mice.

### Detection of C3 Cleavage in the Plasma of Mice by Western Blot (WB)

Plasma was diluted 1/100 in H_2_O. Two volumes of this sample were mixed with one volume of NuPAGE® LDS sample buffer (4X) (Thermofisher) containing reducing agent (DTT 0.33 M) and then denatured at 90°C for 10 min. Proteins were separated in NuPAGE 10% Bis-Tris gel (Thermofisher). The proteins were transferred onto a nitrocellulose membrane using iBlot (Invitrogen). The membranes were incubated overnight with primary antibody (Goat IgG fraction anti-mouse complement C3, MP BIOMEDICALS, #55463), followed by a secondary antibody (rabbit anti-goat HRP, Thermofisher, #31402). Revelation was done by chemiluminescence using a substrate for HRP (SuperSignal® WestDuraLuminol Thermofisher, #1856145), detected by iBright Western Blot Imaging System (iBright FL1500 Thermofisher).

### Statistics

Analyses were performed with GraphPad Prism 8.0. Comparisons of multiple treatment groups were made using one-way analysis of variance (ANOVA) (Dunnett's multiple comparisons test) or Two-way ANOVA Kruskal Wallis test, as indicated in the figure legends. Statistical significance was defined as *p* < 0.05.

## Results

### Pharmacokinetics of Human Hpx in Mice in Presence of Induced Hemolysis

To characterize potential differences in the pharmacokinetics (PK) and the exposure time of Hpx in a mouse model of intravascular hemolysis, induced by phenylhydrazine (PHZ; 0.125 mg/g) two different Hpx doses were administered by bolus intravenous administration through the tail vein (Figure 1A). That allowed the investigation of the PK profile in presence and absence of circulating plasma heme. Blood sampling was performed to cover the range of plasma concentrations from Cmax and to monitor clearance over 72 h in plasma. Figure 1B shows the mean ± SD of human Hpx analyzed at the given timepoint. Pharmacokinetic parameter estimates for human Hpx in absence and presence of hemolysis were calculated via non-compartmental analysis (NCA) and are summarized in Table 1. Upon injection of a lower Hpx dose (100 mg/kg), clearance seemed to be facilitated in presence of circulating plasma heme demonstrated by the decreased half-life (T1/2) of 13.6 h compared to 49.2 h under non-hemolytic conditions. Similar finding, although less pronounced, was observed upon injection of a high Hpx dose (500 mg/kg) with determined half-life of 19.1 h under hemolytic conditions and 41.4 h in absence of plasma heme. Hpx clearance at 100 mg/kg was around 4-fold higher and 2-fold higher at 500 mg/kg in PHZ treated groups compared to PBS groups (Figure 1C).

**Table 1:** Pharmacokinetic parameters of human Hpx in mice (± induced hemolysis).

**Group**	**Dose** **(mg/kg)**	**CL** **(mL/h/kg)**	**Vss** **(mL/kg)**	**C**_**max**_ **(mg/mL)**	**T**_**1/2**_ **(h)**	**AUC**_**0–72 h**_ (h*mg/mL)	**AUC**_**inf**_ (h*mg/mL)
PBS + Hpx	100	2.11	140	1.62	49.2	31.4	47.3
PHZ + Hpx	100	8.13	78.7	1.52	13.6	12.2	12.3
PBS + Hpx	500	2.79	154	7.35	41.4	128	179
PHZ + Hpx	500	4.30	107	7.63	19.1	109	116

*CL, clearance; Vss, volume at steady state; C, concentration; AUC, area under the curve*.

The difference of Hpx pharmacokinetic profiles at the 100 mg/Kg dose, between the PHZ mice pre-treated vs. the non-PHZ pre-treated mice, demonstrated that the Heme:Hpx complexes distribution and elimination were strongly influenced by the complexes affinity to their receptors. This phenomenon was observed as well in the groups of mice treated with a 500 mg/Kg dose of Hpx. However, the difference of Hpx clearance between the non-hemolytic and the PHZ induced hemolytic condition was less important for the 500 vs. the 100 mg/kg Hpx treated mice. This results in a non-linear elimination phase of Hpx in the hemolytic condition, while Hpx terminal elimination phase appears to be linear in the non-hemolytic mice. This demonstrates that in presence of accessible heme in the blood compartment, Hpx pharmacokinetic profile is strongly influence by its affinity to its receptors which is characteristic of a target-mediated drug disposition (TMDD) phenomenon.

### PHZ Induced Hemolysis Increases Total Plasma Heme, Which Is Scavenged by Hpx

In a next study we characterized the different heme binding proteins, especially the presence of Heme:Hpx complexes upon PHZ induced hemolysis. In a similar experimental setup as before, we performed an intravenous injection of human Hpx at two different doses (100 and 500 mg/kg) followed by administration of phenylhydrazine (PHZ; 0.125 mg/g) to induce intravascular hemolysis. Mice were sacrificed at 6 or 72 h after infusion. All mice in the PBS and PHZ-injected group survived (as usual). In the Hpx-injected groups, all mice survived but the dose of 100 mg/kg seems to be well-tolerated at the background of PHZ injection, contrary to 500 mg/kg, for which some mice showed adverse effects. At 6 h 2/10 mice of the 500 mg/kg + PHZ showed weakness, their body temperature was decreased as sensed by the manipulator in comparison to the other mice and had paler paws, nose and ears. At 72 h 2/10 mice showed moderate neurological symptoms (abnormal movement), decreased body temperature and paler paws, nose and ears.

The plasma was analyzed for the presence of total plasma heme, cell free hemoglobin, and Hpx heme complexes. As expected and previously shown (20), total plasma heme increased significantly upon PHZ induced hemolysis assessed after 6 h in all groups in a similar fashion compared to the control group (Figure 2A). In addition, we could demonstrate a significant increase of cell-free Hb, but only in absence of human Hpx (Figure 2B). Dose dependent lower levels of cell free hemoglobin with concurrent dose dependent increase of complexes Hpx (Hpx:heme) at 6 h, as shown in Figure 2C, demonstrates that Hpx scavenges heme from oxidized hemoglobin (metHb). In alignment with the pharmacokinetic behavior of human Hpx in presence of intravascular hemolysis almost no Heme:Hpx complexes were detected after 72 h of infusion (Figure 2D).

### Hpx Partially Prevents Renal Suffering

**Figure 3:**
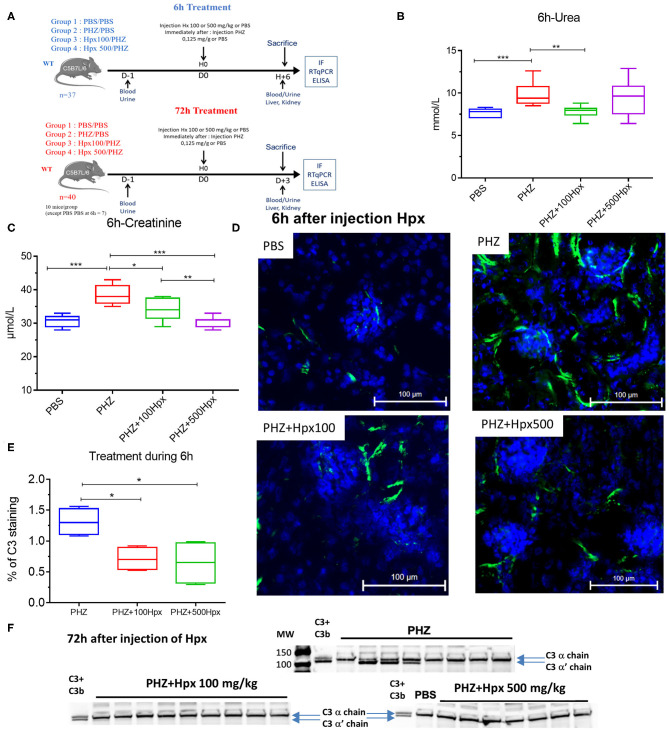
Hemopexin prevents hemolysis-induced complement activation. **(A)** Protocol of mice with or without pretreatment to Hpx in PHZ-treated mice and control **(B)** urea and **(C)** creatinine concentrations in blood 6 h after pretreatment with Hpx or PBS of the PHZ-treated mice, compared to control mice without PHZ treatment measured by KONELAB equipment. **(D)** C3b/iC3b (green) deposition in kidney after 6 h treatment with Hpx to 100 and 500 mg/kg in kidney section **(E)** quantification of C3b/iC3b staining in glomeruli after 6 h treatment with Hpx C3 and CD31 staining were quantified using HALO® (Indica Labs) software. **(F)** Western blot analysis of C3 cleavage in plasma from mice treated with PHZ, PHZ + Hpx 100 and 500 mg/kg and PBS only. Statistical analyses: *p < 0.05, **p < 0.005, and ***p < 0.001, Two-way ANOVA Kruskal Wallis test.

**Figure 4:**
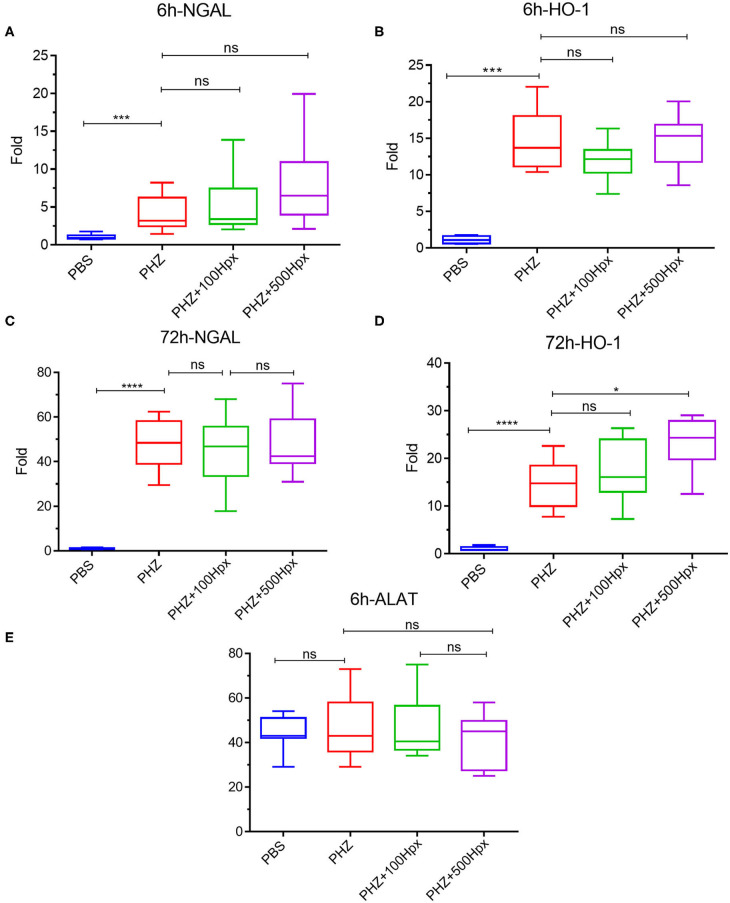
Impact of hemopexin pre-treatment on the kidney parameters (6 and 72 h). Kidney mRNA expression of tubular aggression markers **(A)** LCN2 (NGAL) and **(B)** cytoprotective markers HO-1 statistical analyst 6 h after treatment with Hpx and after 72 h **(C)** LCN2 (NGAL) and **(D)** HO-1. **(E)** ALAT levels in blood 6 h after pretreatment with Hpx on PHZ-treated mice and control. Statistical analyses: *p < 0.05, ****p < 0.0001, Two-way ANOVA Kruskal Wallis test. ns, non significant.

The kidney function was evaluated at 6 and 72 h after induction of hemolysis, in presence or absence of Hpx (Figure 3A). We detected signs of kidney injury in PHZ-treated mice by measuring plasma urea and creatinine levels at 6 h, which were significantly decreased in mice pretreated with Hpx (Figures 3B,C). Further, we studied hypoxic cellular stress response protein Lcn2 (NGAL), a sensitive marker for acute kidney injury. The expression of NGAL in PHZ-treated mice increased compared to PBS controls, but remained unchanged after injection of Hpx (Figure 4A). The upregulation of the cytoprotective enzyme HO-1 by the hemolytic event was also unaffected by the Hpx injection, since injection of Hpx at 100 or 500 mg/kg did not modify expression of HO-1 in PHZ-treated mice (Figure 4B). At 72 h NGAL and HO-1 were still elevated, without effect of the treatment (Figures 4C,D), while urea and creatinine were back to normal (not shown).

No liver injury was detected at the selected time points, since ALAT remained at basal level in presence and in absence of PHZ and Hpx at 6 h (Figure 4E) and at 72 h (not shown).

### Hpx Inhibits the Deposits of C3b/iC3b in the Kidneys of Mice With Intravascular Hemolysis

Since intravascular hemolysis was already evident 6 h after injection of PHZ, we evaluated complement deposits at 6 h in presence and absence of Hpx. We detected a significant increase in C3b/iC3b staining in renal glomeruli within 6 h after inducing intravascular hemolysis with PHZ, which was attenuated by 100 and 500 mg/kg of Hpx (Figures 3D,E). At 72 h the staining was indistinguishable between PBS and PHZ injected mice (data not shown).

### Hpx Inhibits the C3 Activation in Plasma of Hemolytic Mice

Intravascular hemolysis induced by PHZ was associated with C3 activation in the circulation, 72 h after injection. This was evident by the appearance of the α' band on the WB, attesting for appearance of C3b (Figure 3F). The cleavage was heterogeneous. In the PHZ group, among 9 tested plasma samples, the intensity of the α' band was strong in 4; weaker but detectable in 4 and undetectable in 1. Very weak intensity of the α' band was detected in 2/10 tested plasma samples of mice injected with 100 mg/kg Hpx (Figure 3F) and for the remaining 8/10 it was undetectable. In all 8 tested plasma samples of mice injected with 500 mg/kg (Figure 3F), the α' band was absent.

## Discussion

Here we show that during intravascular hemolysis injected Hpx is rapidly complexed with heme and cleared from the circulation, contrary to the context of non-hemolytic mice. The pharmacokinetic characteristics of Hpx were affected by the target-mediated drug disposition phenomenon. Nevertheless, even the lower dose of 100 mg/kg was sufficient to prevent the heme-mediated complement activation in the plasma and in the kidney.

The target mediated drug disposition is a phenomenon in which a drug binds with high affinity to its pharmacological target to such an extent that this affects its pharmacokinetic characteristics (21). The target binding and subsequent elimination of the drug-target complexes could affect both drug distribution and elimination and result in non-linearity of PK in a dose-dependent manner. Our results show formation of Hpx-heme complexes which are rapidly eliminated in the hemolytic mice. This can explain the rapid disappearing of the injected Hpx from the circulation of the PHZ-injected mice, contrary to the control animals. This results in an increase of the effective exposure time to the drug. The target mediated drug disposition and the effective exposure time to the drug, needed to achieve the biological effect are key parameters to be evaluated during the design of therapeutic pre-clinical protocols. Our results provide a rational about the selection of doses to be tested in future experiments.

Increased extracellular concentration of heme is an important driver of the disease state associated with hemolysis. In normal condition the excess of heme is complexed with Hpx and transported to the liver and detoxified. Interestingly, the amount of detected cell-free Hb decreased and the Hpx:heme complexes increased simultaneously when Hpx was injected. This suggests that heme may be taken out from oxidized forms of cell free Hb (MetHb or hemichromes) by Hpx, thus preventing heme to be present in the circulation.

The kidney is one of the most affected organs during intravascular hemolysis (22). In SCD a consumption of Hpx occurs, heme binds to alpha-1-microglobulin, is directed to the kidney and contributes to an acute kidney injury (9). Intravascular hemolysis induces intrarenal complement activation, contributing to the kidney injury (20). Our results show that this complement activation is an early event, detectable at 6 h post-hemolysis but disappearing at 72 h. Moreover, the pre-treatment with Hpx prevented the C3 fragments deposition at the early timepoint, complexing the excess of heme. Even though the concentration of injected Hpx decreased rapidly afterwards, new complement activation did not occur, suggesting that the majority of the cell-free heme was already scavenged. Moreover, new hemolysis did not occur in this model after the initial burst (23). The prevention of complement activation could be attributed to a direct effect of Hpx, preventing the access of heme to C3 (19, 24) and to an indirect, cytoprotective effect, especially on macrophages and endothelial cells (10, 14, 19, 20, 24–27). Glomerular endothelial cells are particularly vulnerable to heme-mediated complement activation in part because they are unable to overexpress HO-1 in hemolytic conditions (23, 28). Therefore, it is likely that Hpx protects glomerular endothelial cells from heme toxicity and they, in turn, do not express complement-activating phenotype. The inflammatory cytokines, released by heme-activated macrophages could also contribute to the endothelial activation and complement deposits, process which also will be indirectly prevented by Hpx. Interestingly, C3 cleavage in plasma was not detected at the early but at the late timepoint. Therefore, the intrarenal C3b/iC3b deposits and the plasma C3 cleavage are separated phenomena occurring consecutively. Although at 72 h there was no more heme release, the tissue injury persisted, as evidenced by the upregulation of the NGAL and HO-1. It is, therefore, tempting to speculate that cell debris released in the circulation from the injured tissues at later timepoints could serve as complement activators in the fluid phase. The cell/tissue protective effect of the heme scavenging even with the lower dose of 100 mg/kg at the early phase of the hemolytic process could explain the fact that Hpx-treated hemolytic mice had no fluid phase C3 cleavage even at a moment, when most of the injected Hpx was already eliminated.

The kidney injury marker NGAL was elevated in hemolytic conditions, which was not prevented by Hpx in agreement with previous studies (23, 29). This result suggests that other factors, such as released Hb and its different oxidation forms or covalently crosslinked Hb multimers or the oxidative stress, hallmarks of intravascular hemolysis (30–33) could be responsible for NGAL upregulation. HO-1 was also up-regulated, but independently of the presence of Hpx, as reported previously (23, 29) and contrary to mice with SCD, where Hpx resulted in further enhancement of the HO-1 expression (11). We hypothesized that in the previous studies the dose of injected Hpx was not high enough to downregulate NGAL and to further enhance the expression of HO-1. Nevertheless, this was not the case, since these makers remained unaltered in our model even at 500 mg/kg Hpx, dose at which Hpx remained in the circulation for the duration of the experiment. Therefore, Hpx could prevent some but not all adverse effects of the hemolytic conditions. Nevertheless, the creatinine and urea were decreased suggesting overall beneficial effect of Hpx on the hemolysis-induced kidney injury. Attention should be made, though, to not exceed the dose of 500 mg/kg for mice with hemolytic conditions. Even though this concentration is very well-tolerated by the control animals, some hemolytic mice showed signs of suffering at this dose.

Based on our data, administration of Hpx could be a possible approach to counteract heme driven toxicity under hemolytic conditions. Therefore, Hpx could potentially be applied as a human blood-derived product similar to other plasma proteins, such as albumin, α1-antitrypsin or immunoglobulins, which are well-established therapies (34). Hpx has already been tested in numerous animal models and showed beneficial effect in most of the tested parameters [(10, 13, 14, 19, 20, 23, 29, 33, 35–41); Table 2].

**Table 2:** Use of Hpx as a therapeutic molecule in different disease model.

**Disease model**	**Animals**	**Used to dose of Hpx**	**Effect of Hpx**	**References**
Rat liver model of cold storage and reperfusion and tested the potential anti-oxidant effects of Hpx	Rat Sprague-Dawley	Reperfused with 5 μM Hpx	Decreasing oxyradical production in a model of cold storage/reperfusion	(35)
Implanted intracranially with 50,000 U87 glioma cells	Nude and BALB/C mice	Intra cerebral delivery to PEX (recombinant Hpx) 0.25–1 with minipumps mg/kg/day (29 days)	Local intracerebral delivery of endogenous inhibitors decreased of tumors growth	(36)
Mesenchymal stem cells-PEX (hMSC-Hpx) injected adjacent to glioblastoma tumors	Nude mice	No dose reported	Mice treated with hMSC-PEX reduction tumor volume and weight measurements decrease 22 days	(37)
SCD and B-thalassemic model	HbS SCD mice and B-thalassemic mice	I.P. To 700 μg injection purified human Hpx	Hpx to treat vasculopathy in hemolytic disorders Decrease cardiac output, aortic valve peak pressure in different mice model	(13)
SCD mouse models	NYDD and Townes SCD mice	I.V. 0.4 or 1.6 μmol/kg	Hemoglobin-induced vaso-occlusion was blocked by the heme-binding protein Hpx	(10)
Hemorrhagic shock (HS) and resuscitated with either FRBCs or SRBCs	C57BL/6 mice	HS and resuscitated with FRBCs/SRBCs to simultaneous infusion of 7.5 mg Hpx	Increase the survival rate and reduced the early proinflammatory response after HS resuscitation with stored blood	(29)
Atherosclerosis	Hpx and Hpx/ApoE KO mice	I.P. human Hpx to HpxApoE KO during 24 h	hHpx significantly reduced serum heme levels Increase in the expression of LXR-α and ABCA1 genes Reduction in expression of CCR-2, and a significant increase in expression of Arg-1	(38)
SCD model	Townes SCD model	I.P. human Hpx (4 mg)	Administration of Hpx is beneficial to counteract heme-driven macrophage-mediated inflammation and its pathophysiologic consequences in sickle cell disease	(14)
Hpx KO and B-thalassemic model	C57BL/6 Hpx^−/−^ and Hbb^th3/+^ mice	160 mg/kg Hpx	Hpx rescued contraction defects of heme-treated cardiomyocytes and preserved cardiac function in hemolytic mice	(33)
Spinal Cord Injury (SCI)	Hpx KO mice	I.P. 0.5–50 ng/mL Hpx	Acute-phase plasma glycoprotein, in the regulation of microglia polarization Hpx in alleviating the secondary injury and improving functional repair after SCI	(39)
Intravascular hemolysis induced by PHZ and heme injection	C57BL/6 and C3^−/−^ mice	I.P. injection of 40 μmol/kg of human Hpx 1 h before heme or PHZ injection	Decreased kidney complement deposition	(20)
Intravascular hemolysis induced by PHZ and heme injection	C57BL/6 mice	I.P. injection of 40 μmol/kg of human Hpx 1 h before heme or PHZ injection	No effect on renal NGAL, Kim1, and HO-1 genes expression	(23)
Cerebral Ischemia reperfusion Injury (CIRI)	Rat Sprague-Dawley	Insert beneath the dural surface to inject rat Hpx (10 μL, 1.86 g/L Hpx)	HPX can alleviate cognitive dysfunction after focal CIRI through HO-1 pathway and preventing the impairment of the blood-brain barrier in rats	(40)
SCD model	Hpx KO and littermate SCD mice Hpx KO (SS Hpx^−/−^ and SS Hpx^+/+)^		Hpx deficiency promotes AKI development in SCD, and we provide proof-of-principle for Hpx replacement therapy to treat AKI in SCD	(41)
Intravascular hemolysis induced by PHZ and heme injection	C57BL/6, C3^−/−^, and TLR4^−/−^ mice	I.P. injection of 40 μmol/kg of human Hpx 1 h before heme or PHZ injection	Decreased NGAL gene expression, decreased liver complement deposition	(19)

In conclusion, hemolysis-induced complement activation is prevented by injection of heme scavenger Hpx. These results encourage further studies of Hpx as a potential therapeutic agent in models of diseases with heme overload, such as SCD, transfusion reactions, etc., taking into account its pharmacokinetic properties.

## Data Availability Statement

The original contributions presented in the study are included in the article/Supplementary Material, further inquiries can be directed to the corresponding author/s.

## Ethics Statement

Experimental protocols were approved by Charles Darwin ethical committee (Paris, France) and of French Ministry of Agriculture (Paris, France) number #3764 201601121739330 v3. All experiments were conducted in accordance with the recommendations for the care and use laboratory animal.

## Author Contributions

LR and GT designed the research. VP, TG, JL, AW, ME, CT, and DL performed research and analyzed data. LR, VP, and TG wrote the manuscript. All authors discussed the data and approved the manuscript. All authors contributed to the article and approved the submitted version.

## Conflict of Interest

TG, AW, ME, DL, and GT are employees of CSL Behring. LR has received research funding from CSL Behring. The remaining authors declare that the research was conducted in the absence of any commercial or financial relationships that could be construed as a potential conflict of interest.

## References

[1] Soares MP Bozza MT. Red alert: labile heme is an alarmin. Curr Opin Immunol. (2016) 38:94–100. 10.1016/j.coi.2015.11.006 26741528

[2] Roumenina LT Rayes J Lacroix-Desmazes S Dimitrov JD. Heme: modulator of plasma systems in hemolytic diseases. Trends Mol Med. (2016) 22:200–13. 10.1016/j.molmed.2016.01.004 26875449

[3] Kanyavuz A Marey-Jarossay A Lacroix-Desmazes S Dimitrov JD. Breaking the law: unconventional strategies for antibody diversification. Nat Rev Immunol. (2019) 19:355–68. 10.1038/s41577-019-0126-7 30718829

[4] Frimat M Boudhabhay I Roumenina LT. Hemolysis derived products toxicity and endothelium: model of the second hit. Toxins. (2019) 11:660. 10.3390/toxins11110660 31766155 PMC6891750

[5] Lin T Maita D Thundivalappil SR Riley FE Hambsch J Van Marter LJ. Hemopexin in severe inflammation and infection: mouse models and human diseases. Crit Care. (2015) 19:166. 10.1186/s13054-015-0885-x 25888135 PMC4424824

[6] Schaer DJ Vinchi F Ingoglia G Tolosano E Buehler PW. Haptoglobin, hemopexin, and related defense pathways—basic science, clinical perspectives, and drug development. Front Physiol. (2014) 5:415. 10.3389/fphys.2014.00415 25389409 PMC4211382

[7] Hvidberg V Maniecki MB Jacobsen C Højrup P Møller HJ Moestrup SK. Identification of the receptor scavenging hemopexin-heme complexes. Blood. (2005) 106:2572–9. 10.1182/blood-2005-03-1185 15947085

[8] Tolosano E Hirsch E Patrucco E Camaschella C Navone R Silengo L. Defective recovery and severe renal damage after acute hemolysis in hemopexin-deficient mice. Blood. (1999) 94:3906–14. 10572107

[9] Ofori-Acquah S Hazra R Orikogbo OO Crosby D Flage B Ackah EB. Hemopexin deficiency promotes acute kidney injury in sickle cell disease. Blood. (2020) 135:1044–8. 10.1182/blood.2019002653 32043112 PMC7218735

[10] Belcher JD Chen C Nguyen J Milbauer L Abdulla F Alayash AI. Heme triggers TLR4 signaling leading to endothelial cell activation and vaso-occlusion in murine sickle cell disease. Blood. (2014) 123:377–90. 10.1182/blood-2013-04-495887 24277079 PMC3894494

[11] Belcher JD Chen C Nguyen J Abdulla F Zhang P Nguyen H. Haptoglobin and hemopexin inhibit vaso-occlusion and inflammation in murine sickle cell disease: role of heme oxygenase-1 induction. PLoS ONE. (2018) 13:e0196455. 10.1371/journal.pone.0196455 29694434 PMC5919001

[12] Ingoglia G Sag CM Rex N De Franceschi L Vinchi F Cimino J. Data demonstrating the anti-oxidant role of hemopexin in the heart. Data Brief. (2017) 13:69–76. 10.1016/j.dib.2017.05.026 28560284 PMC5443894

[13] Vinchi F De Franceschi L Ghigo A Townes T Cimino J Silengo L. Hemopexin therapy improves cardiovascular function by preventing heme-induced endothelial toxicity in mouse models of hemolytic diseases. Circulation. (2013) 127:1317–29. 10.1161/CIRCULATIONAHA.112.130179 23446829

[14] Vinchi F Costa da Silva M Ingoglia G Petrillo S Brinkman N Zuercher A. Hemopexin therapy reverts heme-induced proinflammatory phenotypic switching of macrophages in a mouse model of sickle cell disease. Blood. (2016) 127:473–86. 10.1182/blood-2015-08-663245 26675351 PMC4850229

[15] Camus SM De Moraes JA Bonnin P Abbyad P Le Jeune S Lionnet F. Circulating cell membrane microparticles transfer heme to endothelial cells and trigger vasoocclusions in sickle cell disease. Blood. (2015) 125:3805–14. 10.1182/blood-2014-07-589283 25827830 PMC4490297

[16] Camus SM Gausserès B Bonnin P Loufrani L Grimaud L Charue D. Erythrocyte microparticles can induce kidney vaso-occlusions in a murine model of sickle cell disease. Blood. (2012) 120:5050–8. 10.1182/blood-2012-02-413138 22976952

[17] Roumenina LT Chadebech P Bodivit G Vieira-Martins P Grunenwald A Boudhabhay I. Complement activation in sickle cell disease: dependence on cell density, hemolysis and modulation by hydroxyurea therapy. Am J Hematol. (2020) 95:456–64. 10.1002/ajh.25742 31990387

[18] Vercellotti GM Belcher JD. Not simply misshapen red cells: multimolecular and cellular events in sickle vaso-occlusion. J Clin Invest. (2014) 124:1462–5. 10.1172/JCI75238 24642460 PMC3973116

[19] Merle NS Paule R Leon J Daugan M Robe-Rybkine T Poillerat V. P-selectin drives complement attack on endothelium during intravascular hemolysis in TLR-4/heme-dependent manner. Proc Natl Acad Sci USA. (2019) 116:6280–5. 10.1073/pnas.1814797116 30850533 PMC6442544

[20] Merle NS Grunenwald A Rajaratnam H Gnemmi V Frimat M Figueres M-L. Intravascular hemolysis activates complement via cell-free heme and heme-loaded microvesicles. JCI Insight. (2018) 3:e96910. 10.1172/jci.insight.96910 29925688 PMC6124427

[21] Dua P Hawkins E van der Graaf P. A tutorial on target-mediated drug disposition (TMDD) models: a tutorial on target-mediated drug disposition (TMDD) models. CPT Pharmacomet Syst Pharmacol. (2015) 4:324–37. 10.1002/psp4.41 26225261 PMC4505827

[22] Van Avondt K Nur E Zeerleder S. Mechanisms of haemolysis-induced kidney injury. Nat Rev Nephrol. (2019) 15:671–92. 10.1038/s41581-019-0181-0 31455889

[23] Merle NS Grunenwald A Figueres M-L Chauvet S Daugan M Knockaert S. Characterization of renal injury and inflammation in an experimental model of intravascular hemolysis. Front Immunol. (2018) 9:179. 10.3389/fimmu.2018.00179 29545789 PMC5839160

[24] Frimat M Tabarin F Dimitrov JD Poitou C Halbwachs-Mecarelli L Fremeaux-Bacchi V. Complement activation by heme as a secondary hit for atypical hemolytic uremic syndrome. Blood. (2013) 122:282–92. 10.1182/blood-2013-03-489245 23692858

[25] Lin T Sammy F Yang H Thundivalappil S Hellman J Tracey KJ. Identification of hemopexin as an anti-inflammatory factor that inhibits synergy of hemoglobin with HMGB1 in sterile and infectious inflammation. J Immunol. (2012) 189:2017–22. 10.4049/jimmunol.1103623 22772444 PMC3426910

[26] Pradhan P Vijayan V Gueler F Immenschuh S. Interplay of heme with macrophages in homeostasis and inflammation. Int J Mol Sci. (2020) 21:740. 10.3390/ijms21030740 31979309 PMC7036926

[27] Vinchi F Gastaldi S Silengo L Altruda F Tolosano E. Hemopexin prevents endothelial damage and liver congestion in a mouse model of heme overload. Am J Pathol. (2008) 173:289–99. 10.2353/ajpath.2008.071130 18556779 PMC2438305

[28] May O Merle NS Grunenwald A Gnemmi V Leon J Payet C. Heme drives susceptibility of glomerular endothelium to complement overactivation due to inefficient upregulation of heme oxygenase-1. Front Immunol. (2018) 9:3008. 10.3389/fimmu.2018.03008 30619356 PMC6306430

[29] Graw JA Mayeur C Rosales I Liu Y Sabbisetti VS Riley FE. Haptoglobin or hemopexin therapy prevents acute adverse effects of resuscitation after prolonged storage of red cells. Circulation. (2016) 134:945–60. 10.1161/CIRCULATIONAHA.115.019955 27515135 PMC5039096

[30] Nyakundi BB Erdei J Tóth A Balogh E Nagy A Nagy B. Formation and detection of highly oxidized hemoglobin forms in biological fluids during hemolytic conditions. Oxid Med Cell Longev. (2020) 2020:8929020. 10.1155/2020/8929020 32377310 PMC7196973

[31] Rifkind JM Mohanty JG Nagababu E. The pathophysiology of extracellular hemoglobin associated with enhanced oxidative reactions. Front Physiol. (2015) 5:500. 10.3389/fphys.2014.00500 25642190 PMC4294139

[32] Baek JH D'Agnillo F Vallelian F Pereira CP Williams MC Jia Y. Hemoglobin-driven pathophysiology is an *in vivo* consequence of the red blood cell storage lesion that can be attenuated in guinea pigs by haptoglobin therapy. J Clin Invest. (2012) 122:1444–58. 10.1172/JCI59770 22446185 PMC3314461

[33] Ingoglia G Sag CM Rex N De Franceschi L Vinchi F Cimino J. Hemopexin counteracts systolic dysfunction induced by heme-driven oxidative stress. Free Radic Biol Med. (2017) 108:452–64. 10.1016/j.freeradbiomed.2017.04.003 28400318

[34] Immenschuh S Vijayan V Janciauskiene S Gueler F. Heme as a target for therapeutic interventions. Front Pharmacol. (2017) 8:146. 10.3389/fphar.2017.00146 28420988 PMC5378770

[35] Brass CA Immenschuh S Song D-X Liem HH Eberhard UM. Hemopexin decreases spontaneous chemiluminescence of cold preserved liver after reperfusion. Biochem Biophys Res Commun. (1998) 248:574–7. 10.1006/bbrc.1998.9023 9703968

[36] Giussani C Carrabba G Pluderi M Lucini V Pannacci M Caronzolo D. Local intracerebral delivery of endogenous inhibitors by osmotic minipumps effectively suppresses glioma growth *in vivo*. Cancer Res. (2003) 63:2499–505. Available online at: https://cancerres.aacrjournals.org/content/63/10/2499.long 12750272

[37] Goren A Dahan N Goren E Baruch L Machluf M. Encapsulated human mesenchymal stem cells: a unique hypoimmunogenic platform for long-term cellular therapy. FASEB J. (2010) 24:22–31. 10.1096/fj.09-131888 19726759

[38] Mehta NU Grijalva V Hama S Wagner A Navab M Fogelman AM. Apolipoprotein E ^−/−^ mice lacking hemopexin develop increased atherosclerosis via mechanisms that include oxidative stress and altered macrophage function. Arterioscler Thromb Vasc Biol. (2016) 36:1152–63. 10.1161/ATVBAHA.115.306991 27079878 PMC4882257

[39] Han D Yu Z Liu W Yin D Pu Y Feng J. Plasma hemopexin ameliorates murine spinal cord injury by switching microglia from the M1 state to the M2 state. Cell Death Dis. (2018) 9:181. 10.1038/s41419-017-0236-8 29415995 PMC5833847

[40] Dong B Yang Y Zhang Z Xie K Su L Yu Y. Hemopexin alleviates cognitive dysfunction after focal cerebral ischemia-reperfusion injury in rats. BMC Anesthesiol. (2019) 19:13. 10.1186/s12871-019-0681-2 30646866 PMC6334464

[41] Ghosh S Orikogbo O Hazra R Flage B Crosby D Ofori-Acquah S Hemopexin replacement therapy protects sickle cell disease mice from acute kidney injury. Blood. (2019) 134(Suppl. 1):78. 10.1182/blood-2019-127161

