# Supplementary material for: Hemopexin as an Inhibitor of Hemolysis-Induced Complement Activation
Source: Front Immunol. 2020 Jul 31;11:1684. doi: 10.3389/fimmu.2020.01684 (PMC7412979; doi:10.3389/fimmu.2020.01684)
Supplement: Supplementary file 1 [file Table_1.DOCX]

**Hemopexin as an inhibitor of hemolysis-induced complement activation**

### Victoria Poillerat^1^, Thomas Gentinetta^2^, Juliette Leon^1^, Andreas Wassmer^2^, Monika Edler^2^, Carine Torset^1^, Dandan Luo^3^, Gerald Tuffin^2^, Lubka T. Roumenina^1,*^

**Supplementary Table 1:** Time point to blood samples to determine the pharmacokinetic properties of Hpx in hemolytic mice

| **Time Point-Blood Sample** | | |
| --- | --- | --- |
| *Group 1* | *Group 2* | *Group 3* |
| 15 min | 30 min | 3h |
| 1h | 10h | 48h |
| 6h and sacrificed | 24h and sacrificed | 72h and sacrificed |
